# Evaluation of the Community’s Awareness of Developmental Dysplasia of the Hip: A Cross-Sectional Study in Jordan

**DOI:** 10.7759/cureus.47474

**Published:** 2023-10-22

**Authors:** Farhan H Alkouz, Anas I Kaffaf, Muntaser Abu Shokor, Aimen Abu Qub'a, Amro G Sweedan

**Affiliations:** 1 Department of Orthopaedic Surgery, Ibn Al-Haytham Hospital, Amman, JOR; 2 Department of Orthopaedic Surgery, Speciality Hospital, Amman, JOR; 3 Department of Orthopaedic Surgery, Al-Nadeem Hospital, Ministry of Health, Amman, JOR; 4 Department of Orthopaedic Surgery, Al-Khalidi Hospital, Amman, JOR

**Keywords:** ddh, hip capsule, anatomical abnormality, jordan, developmental dysplasia of the hip, hip joint, awareness

## Abstract

Background: Developmental dysplasia of the hip (DDH) is a condition where there is an abnormal relationship between the femoral head and acetabulum. DDH is prevalent in Jordan, where late presentations of complicated cases are common due to the absence of a national screening program and cultural behaviors that can exacerbate DDH progression. The study aims to prove that the absence of a nationwide screening program and low awareness of the population about DDH are the primary reasons for the high incidence of late-presenting DDH in Jordan. The study highlights the need for addressing modifiable risk factors like swaddling and correcting common misconceptions such as using multiple diapers as a treatment option.

Methods: The cross-sectional study evaluated the awareness level of DDH among Jordan residents above the age of 18. An online questionnaire was used, containing two sections. The Chi-square test was used to analyze the level of awareness scores according to demographic variables and cultural norms such as tight swaddling and using multiple diapers. The sample was categorized into three groups based on their scores: low awareness (0-4), moderate awareness (5-8), and high awareness (9-12).

Results: The study included 1013 participants. The results indicated that 48.9% had moderate awareness, 37.8% had low awareness, and 13.3% had high awareness of DDH. Gender and place of residence had no significant relationship with the level of awareness. However, the majority of participants with high and moderate levels of awareness were in the 25-40 years age group, and healthcare workers had the highest levels of awareness. The study showed that 33.9% of participants gained their knowledge from either relatives or self-experience, while awareness campaigns were the least assessed source of information with only 2.9% of participants getting their information from them. The study also revealed misconceptions regarding DDH, such as the belief that tight swaddling and wearing many diapers reduced the risk of developing DDH. Additionally, about half of the participants were unaware of the presence of screening guidelines for DDH.

Conclusion: The study underscores the urgent need to establish a national screening program and awareness campaigns to increase knowledge about DDH and prevent late presentations of complicated cases in Jordan. The study findings provide essential information for the development of future DDH campaigns and screening protocols.

## Introduction

DDH encompasses a wide spectrum of anatomical abnormalities of the immature hip that manifests in various forms at different stages of life [1]. In 1832, Guillaume Dupuytren described the condition of dislocation of the hip at birth and called it “original or congenital dislocation of the hip” [2].

Ever since it was first described, multiple techniques of reduction have been attempted. However, due to the vigorous nature of the reduction maneuvers, a large number of hips that were treated developed avascular necrosis [3]. Treatment outcomes remained unsatisfactory until 1946 when Arnold Pavlik introduced a harness that became one of the most important advances in the management of DDH. The simple stirrup device allowed for active movement to guide the dislocated hip into the socket. In 1959, he reported the management of 1424 hips without a single case of avascular necrosis [4].

The American Academy of Pediatrics defines DDH as a condition in which the femoral head has an abnormal relationship to the acetabulum [5]. DDH is a disorder that evolves over time. The structures that make up the hip are normal during embryogenesis and gradually become abnormal for a variety of reasons. The etiology of DDH is multifactorial, and it is influenced by hormonal and genetic elements. A number of predisposing factors have been identified, including family history, female gender, firstborn child, ligamentous laxity (often familial), prenatal positioning (especially footling), postnatal positioning (when hips swaddled in extension), and certain ethnic groups [1].

Excessive laxity of the hip capsule is the most common cause of DDH, leading to failure in maintaining the femoral head properly within the acetabulum. DDH is a spectrum disorder where, at one end, it presents as a shallow acetabulum, whereas, at the other end, it involves dislocated and irreducible hips. In between these extremes, it may consist of instability of the hip such that the femoral head can be subluxated or dislocated from the acetabulum by a medical professional. The hip may also present in a dislocated position and still be reducible on examination; however, with chronic dislocation, it may become irreducible with noninvasive management. This disorder may as well manifest later during childhood or adolescence as a dislocated hip or during adolescence as a hip with poorly developed acetabular coverage; the latter being referred to as adult dysplasia of the hip [1].

The incidence of DDH is difficult to determine because of disparities in the definition of the condition, the type of examinations used to detect hip abnormalities, the differing skill levels of examiners, and the populations being studied. The incidence of some degree of hip instability in newborns ranges from one per 1000 to 3.4 per 100. Higher incidences are reported when screening involves both clinical examination and ultrasonography [6]. In addition, the female sex reported two to seven times higher risk [7]. The incidence of DDH for children without associated risk factors is estimated to be 11.5 per 1,000 live births based on meta-analysis protocols and multiple logistic regression [5]. When the risk is calculated for each sex separately, the incidence ranges from 4.1/1,000 for boys to 19/1,000 for girls [8].

According to a study done at the Jordan University Hospital in 2011-2014 regarding the pattern of DDH in late-presenting Jordanian male patients, it was concluded that the incidence of late-presenting DDH among males was 3.75 per 100 infants with a mean age at diagnosis of 4.98 months [9]. On the other hand, a meta-analysis that included a total of 76 studies, most of which were conducted in Europe and Asia including the Middle East, reported a late diagnosis of DDH with the highest incidence of 13 infants per 1000 newborns and the lowest incidence of zero infants per 1000 newborns [10]. Comparing this meta-analysis to the study done in Jordan, it can be deduced that Jordan has an incidence of “late-presenting DDH” in males that is nearly three times greater than the highest incidence elsewhere.

Up until now, there has not been any implementation of screening programs to diagnose DDH in Jordan. The potential early and late-term complications of untreated DDH range from leg length discrepancy to in-toeing or waddling gait, to a variety of hip joint anatomic abnormalities, eventually leading to early onset hip osteoarthritis.

DDH management ranges from noninvasive management using the Pavlik harness or closed reduction and hip spica in younger patients to surgical open reduction and pelvic osteotomy in older patients, with or without femoral shortening osteotomy. Hence, developing a nationwide screening program in Jordan is crucial as it would allow the identification of patients with DDH early. In doing so, we would be able to implement nonoperative management options, which are simple to perform, cheap, and have a high chance of success and a low risk of complications. Therefore, this would reduce the financial and social burden of treating the late presenting DDH and its complications.

Our aim in this study is to prove that one of the major factors leading to such a high incidence of late-presenting DDH in Jordan is the absence of a proper nationwide screening program and the lack of awareness of the population regarding the nature of this disease. The latter includes avoiding some modifiable risk factors such as swaddling and clearing up some common misconceptions such as using two or three diapers as a false treatment option.

## Materials and methods

Study design and participants

This cross-sectional study aimed to assess the awareness level of DDH among Jordan residents. Participants were recruited through convenience sampling via an online self-administered questionnaire that was shared on social media platforms including Facebook (Meta Platforms, Inc., Menlo Park, California, United States), Whatsapp (Meta Platforms, Inc.), and Instagram (Meta Platforms, Inc.). The study was conducted between February and May 2022, and 1013 responses were collected. All study participants were residents of Jordan and over 18 years old.

Ethical considerations

Ethical approval for this study was retrospectively obtained on June 6, 2023, from the Ethics Committee/Institutional Review Board at Ibn Al-Haytham Hospital, Amman, Jordan (approval number: 6.6.2023). Participation was voluntary. The anonymity and confidentiality of the data were ensured by assigning an identification number to each participant restricted to the research team. The questionnaire contained detailed information about the study objectives and returned questionnaires implied signed consent.

Data collection tool

The online questionnaire was designed in English and translated into Arabic. It consisted of two sections. The first section collected data on participants' demographics, including age, gender, marital status, living area, number of children, level of education, and profession. Participants were also asked about their prior knowledge of DDH. If participants reported no prior knowledge of DDH, the questionnaire was automatically submitted at that point and signed as low awareness level. This assumption concluded from a previous study by Alshahrani et al., as they solidify the fact that prior knowledge of DDH is a key factor in understanding the condition and being able to deal with the infant or toddler in a scientific and systematic way, which will decrease the bias for assorting our participants' awareness levels [11].

The second section assessed the awareness level of DDH among participants. It included questions on the source and reliability of information that participants had regarding DDH, as well as their knowledge of risk factors, screening tools, treatment, and complications associated with DDH. The questions in this section were in the form of multiple-choice questions, including direct and indirect questions with correct and incorrect answers. Diagrams and images were also provided to aid in understanding. These questions and answers were referenced from a previous questionnaire conducted in Saudi Arabia, Riyadh, and were reviewed by four pediatric orthopedic doctors [12]. The second section of the questionnaire contained 12 questions, and every right answer was granted 1 mark. The awareness level was calculated based on the total score, with 0-4 indicating low awareness, 5-8 indicating moderate awareness, and 9-12 indicating high awareness. The questionnaire was conducted after a pilot test, followed by face validity by sending the survey to a pediatric orthopedic surgeon.

Statistical analysis

All the analyses have been conducted on R software version 4.2.2 (R Core Team, R Foundation for Statistical Computing, Vienna, Austria) with the proper package for each test. The statistical significance level was set at 0.05. Descriptive statistics were used to examine the demographic characteristics of participants in the sample and to analyze the distributions of responses to questionnaire items and to each scale.

Inferential statistics focus on finding the correlation between different variables. Chi-square test and post-hoc analysis (using the Scheffe test where appropriate) were used to analyze how awareness level scores varied according to different demographic variables. The Chi-square test was also used to assess the relationship between awareness level scores and cultural norms habits (using multiple diapers and swaddling).

## Results

This survey was conducted among 1013 participants in Jordan, with 718 (70.9%) respondents residing in Jordan's capital, Amman; the demographic variables are shown in Table 1. The majority of study participants (47.2%) were between the ages of 25 and 40, and more than half were females (72.2%). In terms of socioeconomic class, 734 (72.5%) respondents held a Bachelor's degree and around 766 (75.7%) were non-healthcare employees. More than half of the respondents (582; 57.5%) were married, while 45% (n=445) did not have children. Of the participants surveyed, 686 individuals (67.7%) indicated that they had knowledge about DDH; these individuals were then questioned further about DDH awareness. The primary source of information for these participants was their relatives or self-experience (33.9%); unfortunately, awareness campaigns were the least common source of information (2.9%).

**Table 1:** Characteristics of study participants DDH: developmental dysplasia of the hip

Characteristics	Variables (n=1013)	Frequency	Percent
Gender	Male	283	27.9
	Female	730	72.1
Age (years)	18-24	261	25.8
	25-40	479	47.2
	41-60	242	23.9
	>60	31	3
Education	Primary School	8	0.8
	Secondary School	176	17.4
	Diploma Degree	78	7.7
	Bachelor Degree	734	72.5
	Master’s degree	12	1.2
	PhD	3	0.3
	Medical Degree	2	0.2
Career Type	Non-medical employment	401	39.6
	Housewife	259	25.6
	Healthcare workers	247	24.3
	Still studying	48	4.7
	Handicrafts and food business	38	3.7
	Not working	20	2
Place of living	Center of Jordan	718	70.9
	South of Jordan	153	15.1
	North of Jordan	142	14
Marital status	Single	374	36.9
	Married	582	57.5
	Divorced	34	3.4
	Widowed	23	2.2
Parity	No children	445	43.9
	1-3	356	35.1
	>3	212	21
Do you know about DDH	Yes	686	67.7
	No	327	32.3

Table 2 shows that nearly half of those who were aware of DDH believed it was caused by medical error (46%) and that female gender and family history are risk factors (46% and 45%, respectively). More than half of them (63.5%) were aware that breach posture is a risk factor for DDH. Interestingly, according to some cultural standards, one-third of respondents were aware that tight swaddling increases the risk of DDH. As expected, over two-thirds (61.4%) of parents employed tight swaddling for their children. Also, nearly two-thirds of the participants (65.6%) felt that wearing many diapers might reduce the incidence of DDH. The majority of respondents (87.1%) believed that more DDH awareness campaigns are needed, and the majority are aware of the potential consequences of DDH and the general management during its early stages. Unfortunately, almost half of our respondents were unaware of clinical or radiological DDH diagnostic methods and ultrasound screening guidelines (33.3%, 49.9%, and 56.7%, respectively).

**Table 2:** Assessment of DDH awareness among the participants DDH: developmental dysplasia of the hip

Questions used to assess DDH awareness	Variables (n=686)	Frequency	Percent
What are the sources that you know about DDH?	Awareness campaigns	20	2.9
	Friends and work colleagues	229	33.4
	Family-doctor	75	10.9
	Self-experience or relatives experience	233	33.9
	Social media	107	15.6
	Study/Work	22	3.2
Do you think that DDH could happen from a medical error?	No (Correct answer)	371	54
	Yes	315	46
What is the gender do you think that associated with higher risk of DDH?	Female (Correct answer)	316	46
	Male	41	5.9
	No difference	329	48
Family history of DDH can contribute to developing a new case of DDH?	No	377	55
	Yes (Correct answer)	309	45
DDH is related to intrauterine position of the baby	Increase by Breach position (Correct answer)	436	63.5
	Increased by Cephalic position	28	4
	No Correlation	222	32.5
What is the risk for swaddling on DDH?	Decrease risk	197	28.7
	Increase risk (Correct answer)	209	30.5
	No effect	280	40.8
What is the risk of use multiple diapers on DDH?	Decrease risk	450	65.6
	Increase risk	71	10.3
	No effect (Correct answer)	165	24
What do you think are the complications of untreated DDH?	Can't walk	33	4.8
	Many complications include (LLD, Limping, Early onset of OA, Chronic pain) (Correct answer)	644	93.9
	No complication	9	1.3
When do you think that DDH can be screened?	12- 18 months of age	2	0.3
	3-6 months of age	204	29.7
	6-12 months of age	19	2.7
	After 18 months of age	3	0.4
	During the first 3 months of age (Correct answer)	458	66.7
When do you think that ultrasound of hip can used to screen for DDH?	After 3 months of age	264	38.5
	During the first 3 months of age (Correct answer)	297	43.3
	No rule for ultrasound	125	18.2
What is the age of the baby that can be screened of DDH using pelvic X ray?	After 6 months of age	97	14.1
	Between 3 - 6 months of age (Correct answer)	344	50.1
	During the first 3 months of age	207	30.2
	No rule for x rays	38	5.6
How DDH is treated during its early diagnosis?	Start with conservative treatment (Correct answer)	673	98.1
	Start with surgical treatment	13	1.8
Do you think that DDH can be prevented?	No (Correct answer)	310	45.2
	Yes	376	54.8
Do you think that we need awareness campaigns?	Need more campaigns	285	41.5
	There are enough campaigns	88	12.8
	There are no campaigns	313	45.6

Following the administration of the questionnaire, participants were assigned to different levels of awareness based on their scores. Those who had not heard of DDH were categorized as having low awareness. We found that almost half of our respondents had moderate awareness of DDH (n=495; 48.9%), more than one-third had a low awareness level of DDH (n= 383; 37.8%), and only 13.3% (n=135) had a high awareness level of DDH. To assess the distribution of awareness levels across demographic characteristics, we utilized the Chi-square test as shown in Table 3. The present study observed no correlation between gender or place of residence and the level of consciousness. There is, nevertheless, a substantial association with other variables including age, education, career type, marital status, and parity (p-value < 0.001).

**Table 3:** Demographic characteristics of participants according to awareness levels

Variables	Total	High knowledge level	Moderate knowledge level	Low knowledge level	P-value
	n=1013	n=135	n=495	n=383	
Age (years)					<0.001
18-24, n (%)	261 (26.6%)	27 (20.3%)	107 (22.3%)	127 (34.3%)	
25-40, n (%)	479 (48.8%)	83 (62.4%)	236 (49.3%)	160 (43.2%)	
41-60, n (%)	242 (24.6%)	23 (17.3%)	136 (28.4%)	83 (22.4%)	
Gender					0.067
Female, n (%)	730 (72.1%)	95 (70.4%)	373 (75.4%)	262 (68.4%)	
Male, n (%)	283 (27.9%)	40 (29.6%)	122 (24.6%)	121 (31.6%)	
Education					<0.001
Below university, n (%)	262 (25.9%)	16 (11.9%)	142 (28.7%)	104 (27.2%)	
University or above, n (%)	751 (74.1%)	119 (88.1%)	353 (71.3%)	279 (72.8%)	
Place of living					0.64
Center of Jordan, n (%)	718 (70.9%)	93 (68.9%)	351 (70.9%)	274 (71.5%)	
North of Jordan, n (%)	142 (14.0%)	24 (17.8%)	70 (14.1%)	48 (12.5%)	
South of Jordan, n (%)	153 (15.1%)	18 (13.3%)	74 (14.9%)	61 (15.9%)	
Career Type					<0.001
Handicrafts and food, n (%)	38 (3.75%)	3 (2.22%)	20 (4.04%)	15 (3.92%)	
Health care workers, n (%)	247 (24.4%)	73 (54.1%)	104 (21.0%)	70 (18.3%)	
Non-Medical employee, n (%)	401 (39.6%)	28 (20.7%)	193 (39.0%)	180 (47.0%)	
Not working, n (%)	327 (32.3%)	31 (23.0%)	178 (36.0%)	118 (30.8%)	
Marital status					<0.001
Single, n (%)	374 (36.9%)	46 (34.1%)	134 (27.1%)	194 (50.7%)	
Married, n (%)	582 (57.5%)	84 (62.2%)	331 (66.9%)	167 (43.6%)	
Widow/Divorced, n (%)	57 (5.63%)	5 (3.70%)	30 (6.06%)	22 (5.74%)	
Parity					<0.001
1 - 3, n (%)	356 (35.1%)	67 (49.6%)	196 (39.6%)	93 (24.3%)	
More than 3, n (%)	212 (20.9%)	17 (12.6%)	128 (25.9%)	67 (17.5%)	
No children, n (%)	445 (43.9%)	51 (37.8%)	171 (34.5%)	223 (58.2%)	

Individuals aged between 25 and 40 comprised the majority of those with high and moderate levels of awareness of DDH, whereas half of those aged between 18 and 25 years had a low level of awareness. Furthermore, most of our respondents who finished high school had higher awareness levels of DDH compared to those who did not complete school education. As predicted, healthcare workers had the highest degree of DDH knowledge when compared to other employment groups. According to marital status and parity, those who were married or had children had the highest level of DDH knowledge (62.2%, each). On the other hand, those who were single or did not have children had the lowest level of awareness (50.7% and 58.2%, respectively).

Our hypothesis was that the self-experience or experiences of relatives with DDH represent the best source of information for achieving a high level of awareness. We tested this hypothesis using a chi-square analysis of the relationship between the level of awareness and the source of information, as outlined in Table 4. As anticipated, individuals with personal experience or those with a family member who has experienced DDH exhibited the highest level of awareness at 37.7%, while the lowest level of awareness was reported among those who had received education from friends or work colleagues, with a percentage of 42.9% and a p-value of 0.007.

**Table 4:** Descriptive summary of chi-square test between source of information and level of awareness

Source of information	Total (n=686)	High knowledge level (n=135)	Moderate knowledge level (n=495)	Low knowledge level (n=56)	P-value
						Friends and work colleagues, n (%)	251 (36.6%)	41 (30.4%)	186 (37.6%)	24 (42.9%)	0.007
Family-doctor, n (%)	75 (10.9%)	26 (19.3%)	42 (8.48%)	7 (12.5%)							
Self-experience or relatives, n (%)	233 (33.9%)	51 (37.7%)	168 (33.9%)	14 (25.0%)							
Social media/awareness campaigns, n (%)	127 (18.5%)	17 (12.6%)	99 (20.0%)	11 (19.6%)							

We examined two crucial cultural norms that may influence the risk of DDH: swaddling practices and the use of multiple diapers. Our objective was to mitigate the incidence of DDH by raising awareness and promoting a reduction in the prevalence of these practices. To this end, we conducted a chi-square test analysis to evaluate the relationship between the level of awareness and the usage of these practices, as presented in Tables 5-6. It is noteworthy that our study yielded interesting results, indicating that individuals with a heightened level of awareness or knowing the potential hazards of swaddling exhibited a significantly lower incidence of swaddling practices, with percentages of 59.3% and 57.9% (p<0.001), respectively. Furthermore, our analysis revealed that increased awareness did not correspond with a higher incidence of multiple diaper usage as a means of mitigating the risk of DDH, with a percentage of 44.4%.

**Table 5:** Descriptive summary of usage of swaddling

Use of swaddling (Yes, No)	Total (n=686)	Yes (n=421)	No (n=265)	P-value
					Awareness level				<0.001
High knowledge level, n (%)	135 (19.7%)	55 (40.7%)	80 (59.3%)	
Moderate knowledge level, n (%)	495 (72.2%)	325 (65.7%)	170 (34.3%)	
Low knowledge level, n (%)	56 (8.16%)	41 (73.2%)	15 (26.8%)	
What is the risk for swaddling				<0.001
Decrease risk, n (%)	197 (28.7%)	178 (90.4%)	19 (9.6%)	
Increase risk, n (%)	209 (30.5%)	88 (42.1%)	121 (57.9%)	
No effect, n (%)	280 (40.8%)	155 (55.4%)	125 (45.6%)	

**Table 6:** Descriptive summary of chi-square test between use of multiple diapers and level of awareness

Use of multiple diapers	Total (n=686)	No effect (n=165)	Decreased risk (n=450)	Increased risk (n=71)	P-value
						Awareness level					<0.001
High knowledge level,n (%)	135 (19.7%)	66 (48.9%)	60 (44.4%)	9 (6.7%)	
Moderate knowledge leveln (%)	495 (72.2%)	92 (18.6%)	349 (70.5%)	54 (10.9%)	
Low knowledge leveln (%)	56 (8.16%)	7 (12.5%)	41 (73.2%)	8 (14.3%)	

## Discussion

The aim of this study was to assess the awareness level of DDH among the population in Jordan. Additionally, the researchers sought to investigate the association between demographic variables and DDH awareness level, with the goal of guiding future awareness campaigns and targeting specific populations. DDH is a common and preventable cause of childhood disability. Unfortunately, delayed diagnosis of the condition increases the likelihood of requiring surgery and raises the risk of long-term complications. To the best of our knowledge, this is the first study to determine DDH awareness in Jordan, serving as a foundation for future research in the field.

In 2018, a study conducted in Riyadh, Saudi Arabia, indicated that approximately 68% of survey participants were already familiar with DDH disease [11]. In a more recent 2022 study in the same location, around 54.4% of respondents reported prior awareness of the condition [12]. In contrast to the Saudi Arabian findings, our study demonstrated that 67.7% of survey participants were already aware of DDH.

When participants were surveyed about the association between breech presentation and the development of DDH, it was found that 63.5% of the participants in Jordan in the present study were aware of this risk factor, whereas only 30.1% in Riyadh, Saudi Arabia, demonstrated the same level of awareness [12]. Similarly, awareness level regarding the increased risk of DDH due to swaddling was reported by 30.5% of participants in Jordan, compared to only 15% in Riyadh. Moreover, the perception that untreated DDH does not result in future complications was indicated by 1.3% of participants in Jordan, whereas the percentage was as high as 6.3% in Riyadh. Regarding the future complication of DDH leading to an inability to walk, 41.7% of participants in Riyadh recognized it as a potential outcome, while only 4.8% in Jordan shared this awareness [12].

Both Jordan and Riyadh demonstrated a higher level of awareness among participants with higher educational levels and those working in the healthcare sector [12]. Conversely, females in Riyadh exhibited a greater understanding of DDH compared to their counterparts in Jordan, where no significant gender-based differences were observed.

It is evident that except for the 18-24-year age group, the majority of the participants had moderate awareness levels regarding awareness of DDH. On the other hand, the majority of participants in the age group of 18-24 years scored low in awareness. Since this is the age group with the highest potential to have children in this society, it is safe to say that they should be the main target of any future awareness campaigns involving DDH.

Linking the level of awareness to the education level of participants our survey also revealed that the percentage of participants with high school level education was significantly lower compared to university level education. That being said the majority of both groups scored in the moderate to low awareness level. As expected, the highest percentage in the high awareness category were the healthcare workers as compared to other professions. However, it is concerning that the majority of the healthcare workers scored in the moderate to low awareness level. It is worth mentioning that the healthcare workers group included nurses, radiology technicians, anesthesia technicians, and nutritionists apart from physicians.

When comparing the level of awareness with marital status, we could see that the majority of the participants, whether single or married, had moderate to low awareness levels. A total of 331 (66.9%) married participants had moderate awareness levels and 194 (50.7%) single participants had low awareness levels when compared to the other marital status subgroups. Additionally, with regard to the level of awareness in relation to the number of children in a family, the majority of participants with children had a moderate awareness level, and those with no children had a low awareness level.

Moreover, our survey’s results revealed that a significant majority of participants relied on personal or familial experiences as well as information from friends as their primary sources of information. In fact, these sources accounted for more than two-thirds of the respondents. Conversely, trusted sources such as physicians and awareness campaigns were unfortunately accessed by only a minority, comprising less than 15% of the respondents. These findings aligned with two studies conducted in Aseer and Riyadh [11,13], both located in Saudi Arabia, which indicated that individuals' awareness of DDH is primarily driven by having a family member affected by the condition.

The main reason behind this behavior can be attributed to the lack of effective awareness campaigns organized by healthcare professionals. This underscores the necessity for such campaigns to enhance people's knowledge and understanding. Hence, it comes as no surprise that more than 86% of the individuals surveyed emphasized the necessity for additional awareness campaigns. Undoubtedly, individuals who had personal experience or witnessed the condition in their relatives possessed the highest level of awareness. These individuals directly receive information about DDH from supervising physicians, which contributes to their heightened knowledge.

Swaddling is a common practice used across many cultures throughout history, in which an infant is wrapped snugly in a cloth. Traditionally, this practice involves tightly swaddling the baby, with the hips in a position of tight extension and adduction [14,15]. Regardless of the other benefits of using traditional swaddling, from an orthopedic perspective, the main concern is the link between the swaddling technique and the incidence of DDH.

The acknowledgment of the adverse impacts associated with conventional swaddling has triggered a transformation in the notion of swaddling that is "hip-safe" [16]. Hip-safe swaddling refers to the practice of swaddling infants while keeping their lower limbs in a position commonly known as the "froggy-leg attitude" [17]. This involves placing the hips in slight flexion and abduction and the knees in slight flexion. Moreover, swaddling in a hip-safe manner should ensure that the infants have enough space to move their hips and knees within the swaddle.

Many previous studies have approved that swaddling and carrying methods for infants that involve forced hip extension-adduction and knee extension are linked to a higher incidence of DDH [18-21]. Conversely, utilizing hip-safe swaddling and baby-wearing devices that allow for unrestricted hip flexion-abduction and knee flexion has been found to promote normal hip development [22-24]. It is of particular importance to recognize the correlation between post-natal positioning and the incidence of DDH, as these risk factors are modifiable.

The results of the current study demonstrate that traditional swaddling is a widespread cultural practice in Jordan, with around two-thirds of participants (n=686, 61.4%) reporting its use. However, this outcome also highlights a lack of awareness among Jordanians regarding the substantial risk that traditional swaddling can pose to the development of DDH. Pinto et al. conducted a recent study in India that aimed to evaluate the level of awareness among healthcare workers (pediatricians, nurses, and caregivers) regarding hip-safe swaddling [25]. The study revealed that only a small percentage of pediatricians (6.6% or three out of 45), caregivers (4% or four out of 100), and none of the nurses (zero out of 219) were knowledgeable about hip-safe swaddling [25]. These findings suggest that there is a low level of awareness about hip-safe swaddling not only among the general population but also among healthcare workers, which calls for increased education and promotion of this important practice.

An effective strategy to decrease the incidence of DDH is through public awareness campaigns that promote the benefits of hip-safe infant positioning. In Japan, an educational campaign was carried out in 1973 to raise awareness of the harmful effects of restrictive lower limb immobilization [15]. A study conducted in 1976 showed a significant reduction in the incidence of DDH in Kyoto, dropping from 52.9 per 1000 births in 1971-1973 to 5.6 per 1000 births in 1974-1976 [26]. The success of the program led to its implementation nationwide, resulting in a decrease in the incidence of DDH in Japan from 3.5% before 1975 to less than 0.2% in 1984 [27]. Encouraging outcomes were also observed in Qatar, where a community program was introduced to raise awareness about the harmful effects of tight swaddling [28]. The program resulted in a reduction of the DDH diagnosis rate from 20% to 6% in selective ultrasound screening.

As demonstrated by the results of this study, increasing awareness about the risks associated with swaddling and DDH can lead to a significant reduction in the incidence of swaddling practices. Additionally, public awareness campaigns aimed at promoting hip-safe positioning have proven effective in decreasing the incidence of DDH in certain populations. Therefore, it is recommended that professional organizations in Jordan, such as the Ministry of Health and the Jordan Orthopedic Association, should collaborate to develop a position statement and a public awareness campaign highlighting the importance of hip-safe swaddling practices and proper techniques for carrying infants, with the goal of reducing the incidence of DDH in the country.

To enhance awareness in Jordan, it is crucial to conduct a series of awareness campaigns across various domains, commencing with high schools, universities, and clinics. These campaigns can effectively encourage parents to detect and address the condition at its early stages. Additionally, to reduce the occurrence of delayed presentation of DDH, it is advisable to establish a screening protocol for early detection or employ an algorithm to identify children who require DDH screening. This approach ensures cost-effectiveness, as it avoids screening all infants for DDH, similar to the screening protocol implemented in southern Australia [29].

This study’s main strength is that it presents a comprehensive assessment of the level of awareness of DDH among Jordan residents using a well-validated questionnaire that enables the reproducibility of the study for future comparison. In addition, this is the first study that measures the level of awareness in Jordan. The limitations encountered in this study include a bias towards respondents residing in urban areas, particularly the capital city. Additionally, our study employed an online questionnaire, enabling us to collect responses from various regions in Jordan. However, this methodology may have influenced the outcomes, potentially leading to relatively similar results among the participants.

## Conclusions

A substantial proportion of participants exhibited a moderate to low level of awareness concerning DDH in Jordan. This knowledge gap is a significant contributing factor to the prevalent issue of delayed DDH presentation. In light of these findings, it is imperative to implement comprehensive nationwide awareness campaigns, focusing primarily on age groups with the highest reproductive potential and high school students. By enhancing awareness levels and rectifying misconceptions surrounding DDH, we can effectively reduce the incidence of delayed DDH presentation, thereby alleviating the burden imposed on the healthcare system in managing both the early and advanced stages of the condition and its complications.
